# T cells in cardiac health and disease

**DOI:** 10.1172/JCI185218

**Published:** 2025-01-16

**Authors:** Pilar Martín, Francisco Sánchez-Madrid

**Affiliations:** 1 Centro Nacional de Investigaciones Cardiovasculares (CNIC), Madrid, Spain.; 2 Centro de Investigación Biomédica en Red de Enfermedades Cardiovasculares (CIBER-CV), Madrid, Spain.; 3 Department of Immunology, IIS Princesa, Hospital Universitario de la Princesa, Universidad Autónoma de Madrid, Madrid, Spain.

## Abstract

Cardiovascular disease (CVD) remains the leading cause of morbidity and mortality worldwide, with inflammation playing a pivotal role in its pathogenesis. T lymphocytes are crucial components of the adaptive immune system that have emerged as key mediators in both cardiac health and the development and progression of CVD. This Review explores the diverse roles of T cell subsets, including Th1, Th17, γδ T cells, and Tregs, in myocardial inflammatory processes such as autoimmune myocarditis and myocardial infarction. We discuss the contribution of T cells to myocardial injury and remodeling, with emphasis on specific immune receptors, e.g., CD69, that have a critical role in regulating immune tolerance and maintaining the balance between T cell subsets in the heart. Additionally, we offer a perspective on recent advances in T cell–targeted therapies and their potential to modulate immune responses and improve clinical outcomes in patients with CVD and in heart transplant recipients. Understanding the intricate interplay between T cells and cardiovascular pathology is essential for developing novel immunotherapeutic strategies against CVD.

## Introduction

T cells are one of the key cellular populations that contribute to cardiac homeostasis and are involved in the development of cardiac diseases. T cells have a dual role in cardiac health. While they safeguard the physiological balance of the heart, they also contribute to the emergence of pathological conditions. T cells are divided into several subtypes. An initial classification comprises CD4^+^ and CD8^+^ T cells. CD4^+^ helper T (Th) cells are further subdivided into Th1, Th2, and Th17 cells and Tregs. Th1 cells produce IFN-γ and mediate the response against intracellular pathogens. Th2 cells secrete IL-4 and IL-13, promoting allergic responses, and provide defense against helminth parasites. Th17 cells produce IL-17 and play a role in autoimmune and inflammatory diseases. Tregs maintain immune tolerance and prevent autoimmunity. CD8^+^ T cells, or cytotoxic T cells, kill virus-infected or cancer-transformed cells. Other subtypes include γδ T cells, which bridge innate and adaptive immunity; and NKT cells, which share characteristics of both NK cells and conventional T cells that contribute to immune regulation and response.

In this Review, we begin by defining the T cell subsets and their known roles in myocardial and cardiovascular disease (CVD). We then describe the functions of heart-specific T cell receptors (TCRs) and immunoregulatory receptors, focusing on the activation molecule CD69, whose ligands may represent therapeutic targets for cardiac and cardiovascular conditions. We also assess the various T cell subtypes that infiltrate the myocardium under both healthy and pathological conditions, as well as the distinct types of specific receptors and adaptive immune responses that occur during homeostasis and in various cardiac diseases. Finally, we discuss the contribution of T cells to myocarditis, cardiotoxicity, and cardiac fibrosis, as well as the potential for immunotherapies to be leveraged in cardiac conditions and cardiovascular disease.

## T cell subsets in myocardial homeostasis and damage

**Figure 1:**
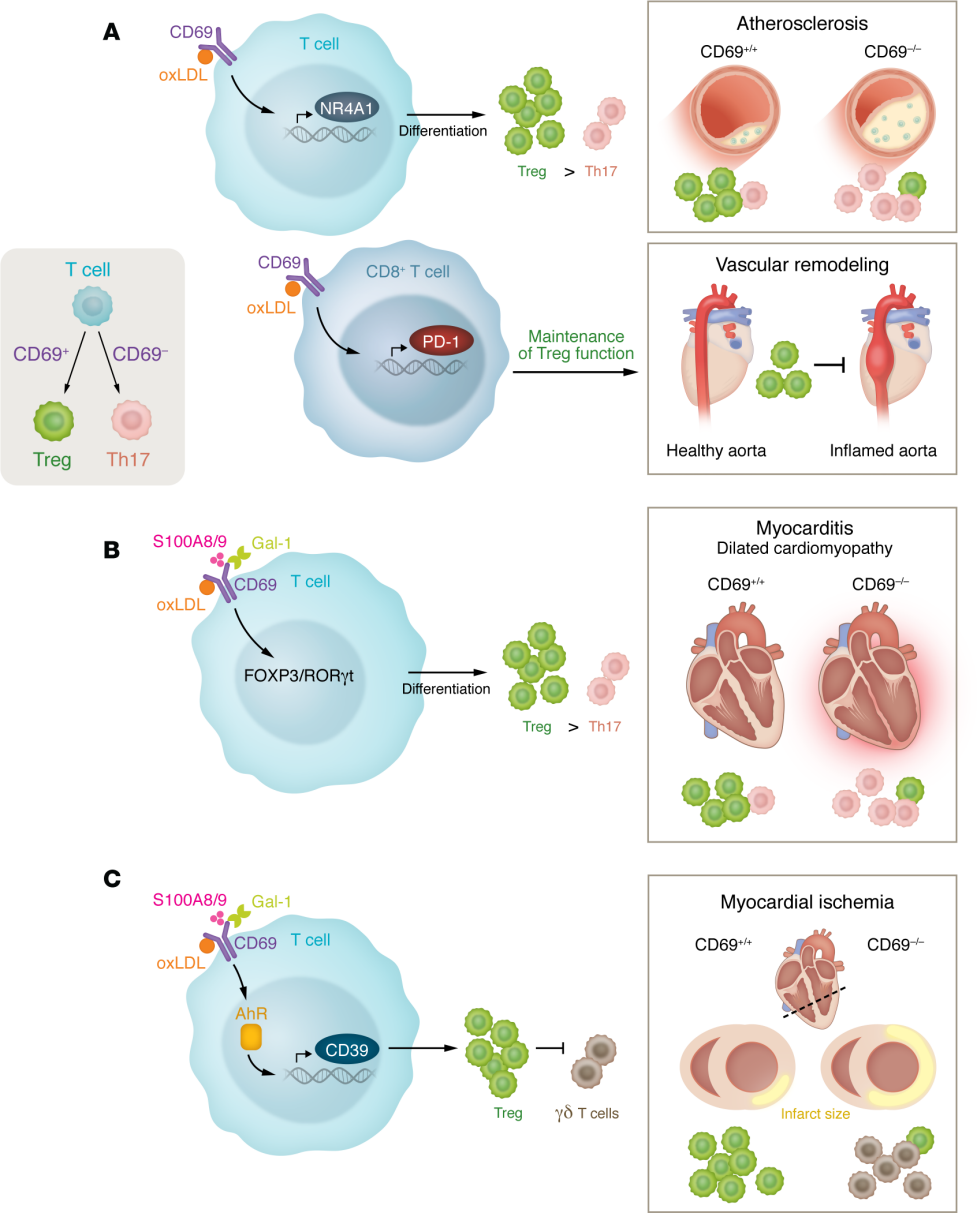
The role of CD69 in various cardiovascular pathologies. (**A**) oxLDL binding to the CD69 receptor regulates NR4A expression in T cells, which has been shown to promote Treg differentiation. In mice, CD69 deficiency has been linked to altered NR4A1 expression, Treg–Th17 cell imbalance, and exacerbation of atherosclerosis. oxLDL/CD69 signaling also regulates PD-1 expression in CD4^+^ T cells, which is known to regulate vascular changes in the inflamed aorta. (**B**) The CD69 receptor’s interaction with oxLDL, Gal-1, and S100A8/S100A9 regulates the FOXP3/RORγt pathway to promote Treg differentiation. In models of myocarditis and dilated cardiomyopathy, CD69^–/–^ hearts have altered Treg–Th17 cell immune cell infiltration and altered RORγt/Foxp3 signaling. (**C**) In models of myocardial ischemia, CD69 deficiency increases infarct size. CD69 is linked to activation of the aryl hydrocarbon receptor (AhR) and increased CD39 transcription, which promotes Treg control of γδ T cell activity.

Recent single-nucleus RNA sequencing (snRNA-Seq) (1) and single-cell RNA sequencing (scRNA-Seq) (2, 3) studies have identified diverse T cell populations within the myocardium, including CD4^+^ helper T cells, CD8^+^ cytotoxic T cells, Tregs, γδ T cells, and NKT cells, that contribute to a balanced immune environment. The presence of these diverse T cell populations ensures that the heart remains protected from infections and inflammation, highlighting their critical role in the overall function and resilience of healthy cardiac tissue (Figure 1).

T cells play a critical role in ischemic damage, contributing to the initial injury response and subsequent repair processes. Following ischemic events such as myocardial infarction (MI) (4, 5), T cells are rapidly recruited to the damaged myocardium. T cells infiltrate the myocardium following ischemia/reperfusion. CD4^+^ T cells, but not CD8^+^ T cells, contribute to myocardial ischemia/reperfusion injury (MIRI) by recognizing self-antigens (6). T cell–deficient mice retained left ventricular (LV) function, reduced fibrosis, hypertrophy, and inflammation; and showed improved survival compared with WT mice. Depleting T cells in WT mice after constriction prevented heart failure (HF), indicating that T cell activation and infiltration into the LV are critical in HF progression, likely through cytokine release and induction of cardiac fibrosis and hypertrophy (7). CD4-knockout and OTII TCR-transgenic mice displayed a smaller region of infarction compared with WT mice, demonstrating the detrimental role of CD4^+^ T cells. These mice had less neutrophil infiltration in the affected area, underscoring the involvement of CD4^+^ T cells in ischemic damage (4).

Tregs, on the other hand, modulate the inflammatory response, promoting tissue repair and regeneration. MIRI significantly impacts the final infarct size in acute MI (AMI). The local inflammatory microenvironment influences healing after AMI, particularly due to the activity of Tregs, which reduce MIRI, thereby improving patient prognosis. Different Treg subtypes have various effects on MIRI, and their impact can change at different stages of MIRI (8).

NKT cells appeared to improve the outcome in an acute model of MI in mice (9). Mice with induced MI treated with the prototype antigen α-galactosylceramide (α-GC), which is presented by CD1d and recognized by NKT cells, displayed longer survival and less heart fibrosis compared with vehicle-treated mice. The number of NKT cells increased following α-GC treatment and correlated with IL-10 expression. Blocking IL-10 impaired the beneficial effects of α-GC, suggesting that the functional role of NKT cells in MI is mediated by the antiinflammatory actions of IL-10 (9).

The role of γδ T cells in the heart is controversial. For example, γδ T cells protect the heart in a mouse model of Duchenne muscular dystrophy (DMD) by killing pathogenic macrophages and potentially delaying cardiac damage (10). Conversely, studies of enterovirus infections, which are a trigger of myocarditis and dilated cardiomyopathy (DCM), show that myocarditis susceptibility depends on γδ T cell activation (11): Only mouse strains with γδ T cells in the myocardium exhibited myocyte apoptosis or DCM-like disease (12). In this study, infected myocytes showed increased Fas expression, leading to Fas-mediated apoptosis. Approximately 38% of CD3^+^ lymphocytes in the heart are γδ T cells. These cells triggered more Fas-mediated myocyte apoptosis than αβ T cells, implicating them in myocardial injury during viral myocarditis (12). Likewise, γδ T cells are involved in the pathogenesis of coronary atherosclerotic heart disease: In a study of 25 patients with AMI, mRNA expression levels of TCR Vγ1, Vγ2, and Vγ3 subfamilies were significantly higher than in healthy control individuals (13). AMI patients also exhibited restricted TCR Vδ subfamily expression, with lower frequencies of TCR Vδ6 and Vδ7 and greater clonal expansions of TCR Vδ3, Vδ4, and Vδ8. High IL-17A expression was found in AMI γδ^+^ cells. These findings suggest that γδ T cells display a distinctive TCR γδ repertoire and altered *IL-17A* gene expression in AMI, potentially contributing to the immune response and worsening the clinical outcome of patients with AMI (13).

Tissue-resident memory T cells (Trm cells) are found in the mouse myocardium in homeostasis (14) and in aging (15), and in pathological conditions in humans and mice (16). Trm cells have been shown to contribute to local immune surveillance and rapid response to infections, thus playing a key role in protecting cardiac tissue from infection and inflammation (17–19). A small subset of Trm cells has been identified in human atherosclerotic lesions, with a phenotypic signature characterized by expression of CD69 and the integrin CD49a, and were associated with increased lesion stability in a mouse model (14, 20). These findings suggest that Trm cells play a critical role in maintaining immune surveillance and modulating inflammation in the heart, under both normal and pathological conditions, potentially contributing to cardiovascular health and disease progression.

## TCR-specific responses and autoimmune damage

TCR-specific responses to cardiac self-antigens trigger autoimmune damage to the heart, specifically myocarditis and DCM (21–24). α–Myosin heavy chain (α-MyHC), a component of the thick filament in myocytes, was used as a primary autoantigen for CD4^+^ T cells in a spontaneous mouse model of myocarditis (25). Because α-MyHC transcripts are absent in mouse medullary thymic epithelial cells (mTECs) and peripheral lymphoid stromal cells, mice (and humans) do not develop T cell tolerance against these antigens (26). Transgenic expression of α-MyHC in murine thymic epithelium conferred tolerance to cardiac myosin and prevented myocarditis, suggesting that impaired central tolerance to α-MyHC contributes to myocarditis. This study corroborated these findings in humans, in which patients with myocarditis showed elevated frequencies of α-MyHC–specific T cells in peripheral blood (27). In addition, the abundant expression of MYH6-specific TCR, as described previously (28), further underscores the role of T cells in cardiac autoimmunity. Cross-reactivity with commensal mimic peptides, as highlighted by a recent study (29), can exacerbate the activation of MYH6-specific T cells, leading to enhanced autoimmune responses. This cross-reactivity may provide a mechanistic explanation for the activation of autoreactive T cells and the progression of myocarditis, contributing to a broader understanding of the loss of immune tolerance in the heart.

Patients with myocarditis and DCM displayed increased numbers of CD4^+^Th17^+^ T cells, which in turn produced higher amounts of Th17-related cytokines (IL-6, TGF-β, IL-23). Persistent HF was associated with high levels of IL-17–producing T cells and cytokines together with low percentages of FOXP3^+^ Tregs. Cardiac myosin–derived TLR2 ligands exacerbated Th17-related cytokine production by myocarditis monocytes, which was inhibited by an anti-TLR2 antibody (30).

Understanding the specific TCR-mediated mechanisms and the nature of cardiac antigens involved in these responses is vital for developing therapies aimed at preventing or mitigating autoimmune cardiac diseases while preserving necessary immune functions.

## Immune-regulatory receptors in cardiac T cells

In this Review, we focus on CD69 due to its critical role in modulating immune responses, particularly in the cardiovascular system. Work from our group and others has demonstrated that CD69 is a key regulator of T cell responses, including the balance between proinflammatory Th17 cells and Tregs, which are crucial in the pathogenesis of CVDs such as myocarditis, MI, and atherosclerosis. CD69’s interaction with different ligands has been shown to modulate immune activity, suggesting its potential as a therapeutic target to prevent inflammation-driven cardiac damage. This body of research supports exploration of CD69 as a promising target for future immunomodulatory therapies in cardiovascular conditions.

### CD69 is a regulatory receptor of inflammatory CVD.

The leukocyte activation marker CD69 was identified by several groups as an early activation molecule. CD69 (termed EA-1, AIM [activation inducer molecule], or Leu23 in these early studies) was found to be expressed by leukocytes during activation (31–33) and constitutively by Trm cells (16). CD69 expression is undetectable in vivo in resting peripheral blood lymphocytes, but it is expressed in inflammatory cell infiltrates of various chronic inflammatory diseases (34–36). Biochemically, the CD69 molecule is a C-type II lectin disulfide-linked homodimer (24 kDa) containing a carbohydrate recognition domain (CTLD) at its C-terminal end (37–39). The *CD69* gene is in the long arm of mouse chromosome 6, syntenic of chromosome 12 in humans. *CD69* is within the ‘‘NK complex’’ region, which comprises several genes of the family of C-type lectins specific for NK cells (37, 40, 41). The crystal structure of CD69 CTLD has been solved. Like other NK cell receptors of the same family, it mainly binds carbohydrates, although it also has the potential to bind protein ligands (42). CD69 is induced transcriptionally, and the transcription factors involved in its expression have been described in detail elsewhere (43). CD69 ligation using specific ligands or antibodies triggers an elevation of intracellular Ca^2+^ levels and ultimately drives T cell activation and proliferation (44).

### CD69 and T cell differentiation.

CD69-deficient mice appear normal, with no apparent developmental defects (45). CD69-deficient mice bred in a C57BL/6 background with OTII mice (which generate OVA-specific CD4 T cells, making them a key model for studying CD4^+^ T cell activation and immune responses) and triggered with OVA revealed no significant differences in Th1 and Th2 cell–specific cytokines. In contrast, IL-17 secretion was increased in Th1, Th2, and Th17 cells. Th17 cell transcription factor RORγt and phospho–STAT-3 levels were also elevated, consistent with enhancement of the Th17 cell differentiation potential of CD4^+^ T cells in CD69-deficient mice (46). Proteomic analysis revealed that the C-terminus tail of CD69 interacts with STAT5 and JAK3 (46), constituting a potential mechanism by which CD69 may inhibit Th17 cell differentiation. CD69 also controls immune tolerance by regulating the suppressor activity of Foxp3^+^ Tregs and thymic development of Tregs. This seems to depend on the regulation of BIC/microRNA-155 (miR-155) and its target, suppressor of cytokine signaling 1 (SOCS-1) (47, 48). CD69 acts as a key regulator of Treg development and homeostasis. Hence, CD69-activated STAT5 antagonizes STAT3-mediated RORγt activation and activates the transcription factor FoxP3, which in turn stimulates the differentiation of Tregs. Through this mechanism, CD69 appears to control the immune response balance of Tregs and Th17 cells (49).

### CD69 in inflammatory and cardiovascular responses in vivo.

Due to the pivotal role of CD69 in T cell biology, CD69-deficient mice have been used to generate new insights about the role of T cells in cardiovascular health and disease (Figure 1). The regulatory role of CD69 in the inflammatory response was first examined in a murine model of collagen-induced arthritis (CIA). CD69-deficient mice developed an exacerbated form of CIA, with significantly higher incidence and severity compared with WT mice. Arthritic joints displayed reduced levels of TGF-β1 mRNA and increased levels of IL-1β and RANTES, which may account for the enhanced inflammatory response observed in CD69-deficient mice (50). These studies have been extended to other models of chronic inflammation with Th17 involvement, such as asthma, contact hypersensitivity, and inflammatory bowel disease, the latter mainly during the sensitization phase through the regulation of Ag-specific effector T cells (51–54).

IL-17 plays a pivotal role in the pathogenesis of experimental autoimmune myocarditis (EAM), which models inflammatory heart disease (55). IL-17 is produced primarily by Th17 cells and has been implicated in driving inflammation and autoimmune responses in the heart (56, 57). Elevated levels of IL-17 in EAM are associated with increased recruitment of neutrophils and other inflammatory cells to the myocardium, leading to tissue damage and exacerbation of the disease. Additionally, IL-17 promotes production of other proinflammatory cytokines and chemokines, further amplifying the inflammatory response (58). Studies have shown that neutralizing IL-17 or inhibiting its signaling pathways can reduce the severity of myocarditis and improve cardiac function in animal models (59, 60) In EAM, CD69 negatively regulated cardiac inflammation through control of heart-specific Th17 responses (61) (Figure 1). On the other hand, CD69 controlled chronic cutaneous inflammation in psoriasis by regulating Tγδ and CD4^+^ Th17 cell responses (62). Taken together, these studies underscore that CD69 acts as an inhibitor that controls the extent of local immune responses, thereby playing an important role in the suppression of inflammatory disease.

### CD69 ligand interactions: potential targets in CVD.

The search for protein CD69 ligands can be divided into cellular and extracellular ligands (Figure 1). Regarding the former, recombinant chimeric proteins bound to human immature monocyte-derived DCs (iDCs) as well as primary Langerhans cells (LCs) were immunoprecipitated and subjected to mass spectrometry. This approach identified galectin-1 (Gal-1) as a potential CD69 ligand, which was confirmed by surface plasmon resonance (63). The interaction of Gal-1 with CD69 decreased mRNA levels of the Th17 cell transcription factor RORC2, as well as the number of IL-17–producing cells, and inhibited Th17 cell differentiation. In contrast, these effects were not observed in CD69-deficient cells. Thus, it became apparent that Gal-1 interaction with CD69 modulates the differentiation of Th17 lymphocytes. The relevance of Gal-1 in pathological vascular remodeling was underscored by the finding that Gal-1–deficient mice displayed increased atherosclerotic burden and instability compared with WT littermates in a model of inducible severe atherosclerosis (64).

The S100A8/S100A9 complex is another CD69 ligand. CD69 binding to S100A8/S100A9 depends on their carbohydrate moieties (Figure 1). This complex also controls the Treg/Th17 cell differentiation balance (65). The interaction is also important during monocyte migration and the regulation of their inflammatory status (66). Similar approaches have identified myosin light chain 9 and 12 as functional ligands for CD69 in activated helper T lymphocytes, including a role for this interaction in airway inflammation and in inflammatory bowel disease (67, 68).

Oxidized LDL (oxLDL) is a key antigen that activates T cells in the atherosclerotic lesion, contributing to the chronic inflammation seen in plaque development. ApoB, the main protein component of LDL particles, is another autoantigen that can stimulate T cell responses. Additionally, heat shock proteins, which are upregulated during cellular stress, are also involved in the autoimmune response, in which they contribute to atherosclerosis. Among the noncellular ligands of CD69, apolipoproteins ApoE and ApoB and oxLDL were identified by mass spectrometry. Both types of proteins are key elements in the development of atherosclerosis. Interestingly, oxLDL seems to influence adaptive immune responses, but their putative receptor on T lymphocytes has remained elusive. The main receptor of oxLDL on vascular cells is lectin-like oxLDL receptor 1 (LOX-1) (69), which has a 3D structure strikingly similar to that of CD69 (70). The oxLDL-binding surface of LOX-1 contains a diagonal arrangement of arginine residues on the top part of the dimer, and a similar spine-like arrangement can be found in the CD69 dimer. Activated primary T cells and cells stably expressing CD69 on their surface bound oxLDL much more efficiently than control cells (70). Functionally, treatment of human CD4^+^ T cells with oxLDL diminished the percentage of IL-17^+^ and IFN-γ^+^ cells generated in response to Th17- and Th1-polarizing stimuli, respectively, and favored Treg differentiation in a CD69-dependent manner (70). Mechanistically, oxLDL increased expression of the antiinflammatory transcription factors NR4A1 and NR4A3 mRNA in activated T cells (70). Remarkably, mice bearing CD69-depleted lymphoid cells that were subjected to a high-fat diet displayed a Th17 cell/Treg imbalance, with exacerbated atherosclerosis (70). Additionally, CD69 promotes programmed cell death 1 (PD-1) expression in CD4^+^ T lymphocytes after engagement with oxLDL, which may be responsible for the exacerbated activation state found in the absence of CD69 in an atherosclerosis model (71) (Figure 1).

### Other CD69-associated proteins.

CD69 can control lymphocyte migration and egress from the lymph node by its association with and regulation of membrane expression of the sphingosine 1 phosphate receptor 1 (S1P1) (72, 73). CD69 acts as a *cis* ligand of S1P1, preventing CD69^+^ T cells from exiting lymphoid tissues (74). This interaction plays a critical role in regulating T cell circulation and maintaining immune homeostasis, which is crucial for both cardiac and systemic immune responses. Likewise, a similar role has been shown for CD69 on DCs as they migrate to lymph nodes (75). CD69 also interacts with the amino acid transporter complex SLC7A5-SLC3A2, playing a role in the complex’s stability and uptake of Leu and Trp on the plasma membrane of T lymphocytes (62, 76), which is important for regulation of Th17 and γδ T cell subsets and their function in skin inflammation (77).

### CD69 in acute MI.

CD69^+^ Tregs reduce inflammation and improve survival after MI. In mice, CD69 deficiency worsened cardiac function, increased myocardial damage, and reduced survival. Indeed, CD69+ Tregs inhibited IL-17–producing γδ T cells through CD39-dependent mechanisms, reducing inflammation and cardiac damage (Figure 1). Clinical data from patients showed that higher CD69 expression in Tregs after MI correlated with a lower risk of rehospitalization for HF. These findings suggest that enhancing CD69 expression in Tregs could be a potential therapeutic strategy to improve the outlook for patients with MI (78).

## Circulating c-Met–expressing memory T cells in cardiac autoimmunity

A subset of c-Met^+^ memory T cells that preferentially migrate to cardiac tissue in patients with inflammatory cardiomyopathies display preferential proliferation in response to cardiac myosin and production of multiple cytokines, such as IL-4, IL-17, and IL-22. The presence of c-Met^+^ T cells in the blood and myocardium of patients with acute myocarditis (AM) and idiopathic DCM (iDCM) signals the onset of an adaptive immune response targeting the heart. In EAM, pharmacological inhibition of c-Met reduced disease severity in mice, implicating c-Met^+^ T cells in the pathogenesis of cardiac autoimmunity (79). The study suggests that c-Met^+^ T cell levels could serve as a diagnostic and prognostic marker for myocardial inflammation and provides a potential therapeutic target for treating inflammatory heart diseases (79).

## T cells and cardiotoxicity

Cardiotoxicity is a frequent side effect of chemotherapeutic agents and immune therapies. It involves damage to the muscle tissue of the heart, which can lead to serious cardiovascular complications. Activated T cells can infiltrate the myocardium, causing inflammation that damages cardiac cells. This is particularly evident in immune checkpoint inhibitor therapy, in which enhanced T cell activity intended to target cancer cells also results in unintended cardiac injury. The interaction of T cells with cardiac antigens can evoke an autoimmune response, exacerbating cardiotoxicity (80, 81). In a study of fatal myocarditis in patients with melanoma who were treated with a combination of the immune checkpoint inhibitors ipilimumab (targeting CTLA-4) and nivolumab (targeting PD-1), two patients developed severe myocarditis and myositis, accompanied by extensive T cell and macrophage infiltration in the myocardium and skeletal muscle. Both patients exhibited rapid clinical deterioration despite receiving treatment with high-dose glucocorticoid. Histopathological analysis revealed identical T cell receptor sequences in infiltrates from cardiac, skeletal muscle, and tumors, consistent with shared antigen recognition. Although MYH6 and troponin are primarily expressed in cardiac tissue, there is evidence that aberrant expression of cardiac antigens can occur in tumors, leading to cross-reactivity in immune responses (68). Further analysis showed increased expression of inflammatory cytokines and muscle-specific transcripts in tumor specimens. These findings underscore the potential for immune checkpoint inhibitors to cause severe, potentially fatal myocarditis, highlighting the need for specific monitoring and suggesting that T cells are central mediators of this adverse effect (82).

The role of CD8^+^ T cells in immune checkpoint inhibitor–associated myocarditis (ICI-MC) has been addressed using a *Pdcd1^–/–^* *Ctla4^+/–^* mouse model that mimics human ICI-MC. Single-cell RNA and TCR sequencing revealed that clonal effector CD8^+^ T cells are prevalent in myocardial immune infiltrates (Figure 1). The study identified α-myosin, a heart-specific, protein as a crucial autoantigen. Peripheral blood T cells from patients with ICI-MC, when expanded by α-myosin peptides, shared TCR clonotypes with those in diseased heart and muscle tissue, indicating the clinical relevance of α-myosin. Depleting CD8^+^ T cells, but not CD4^+^ T cells, improved survival. Adoptive transfer experiments confirmed that CD8^+^ T cells are necessary for fatal myocarditis. These findings highlight the central role of cytotoxic CD8^+^ T cells in ICI-MC and identify α-myosin as a cause of this adverse effect and thus a potential therapeutic target (83).

## T cell–fibroblast intercellular communication

The interaction between T cells and fibroblasts is important in the pathophysiology of various CVDs. In the context of cardiac injury, activated T cells can infiltrate the myocardium and interact with resident fibroblasts (84). Cardiac fibroblasts express major histocompatibility complex class II (MHC II) and function as antigen-presenting cells (APCs) during cardiac inflammation, activating CD4^+^ T cells by in situ antigen presentation (85–87). This interaction often leads to secretion of proinflammatory cytokines and growth factors, which in turn activate fibroblasts and promote their differentiation into myofibroblasts (88). These myofibroblasts mediate an excessive deposition of extracellular matrix components, leading to fibrosis and impaired cardiac function (7). T cell activation and differentiation are regulated in specialized niches supported by fibroblasts in lymphoid organs and inflamed tissues (89). In the healthy heart, fibroblastic stromal cells make up about 20% of non-cardiomyocytic cells. During inflammation, fibroblast activation affects T cell functionality in the diseased cardiac microenvironment (90). Dysregulated bone morphogenetic protein (BMP) signaling in fibroblasts attempts to maintain homeostasis in the heart during T cell and macrophage-dominated myocardial inflammation. Fibroblasts are the primary source of BMP4, but its expression is reduced by inflammatory mediators such as IL-1β. BMP4 downregulation is associated with a switch of cardiac fibroblasts from a homeostatic to an inflammatory state, including T cell recruitment and loss of cardiomyocyte integrity (91, 92). These findings underscore the importance of the interaction of T cells with other cells in the cardiac niche and the inflammatory milieu in maintaining cardiac homeostasis and controlling myocardial inflammation.

## Therapy opportunities

While immunotherapy to target immune checkpoints and modulate immune responses has revolutionized oncology, its applications in CVD are underexplored. Below, we discuss each immunotherapeutic modality and its potential for treating cardiac and cardiovascular conditions.

### Antibodies, cytokines, and immunotherapy.

A promising avenue for application of immunotherapy to CVD is modulating T cell effector responses and promoting Treg activity (93, 94). Intravenous administration of anti-CD3–specific antibodies effectively suppresses effector T cell immune responses and reduces atherosclerosis in mice by inducing TGF-β–producing CD4^+^CD25^+^ LAP^+^ Tregs, which in turn inhibit experimental autoimmunity in a TGF-β–dependent manner (95, 96). Combining anti-CD3 antibodies with IL-2 complexes increases Treg numbers and halts the progression of atherosclerosis in ApoE^–/–^ mice (97). On the other hand, CD147 inhibition blocks T cell activation and immune cell recruitment to the heart in CVB3-induced myocarditis (98).

Cytokine-based immunotherapies can modulate immune responses, aiding in cardiac repair for patients with HF (99). IL-37, a potent antiinflammatory cytokine that belongs to the IL-1 family, has shown promise for treatment of CVD (100–104). A recent study revealed that IL-37 levels were lower in patients with coronary artery disease (CAD) than in healthy volunteers. Also, IL-37 inversely correlated with inflammatory markers, thus becoming a predictor of CAD, which suggests that its decreased levels in CAD patients are linked to inflammation and disease progression (105). Another clinical study revealed that IL-37 is elevated in patients with acute coronary syndrome, having a beneficial role (106). Recombinant IL-37 administration increases the release of the antiinflammatory cytokines IL-10 and TGF-β from Tregs in vitro. These findings suggest that recombinant IL-37 could mediate antiinflammatory effects in atherosclerosis by enhancing Treg function and cytokine secretion (107).

The CANTOS, LoDoCo, and COLCOT trials have confirmed that targeting inflammation can improve cardiovascular outcome in atherosclerosis (108–110). These studies demonstrated that inhibiting IL-1β and neutrophil function with colchicine reduces the progression of atherosclerotic CVD. Given the important role of adaptive immunity, particularly T cell activation, in atherosclerosis, immune checkpoint inhibitors may also offer important therapeutic benefits for managing the disease.

Th1 cells are the main CD4^+^ T cells contributing to atherogenesis through their production of IFN-γ and TNF-α. IFN-γ enhances recruitment of macrophages and T cells; promotes macrophage polarization, cytokine secretion, and foam cell formation; and inhibits vascular smooth muscle cell proliferation, leading to decreased plaque stability (111, 112). TNF-α contributes to atherosclerosis by recruiting leukocytes, producing inflammatory cytokines, and causing endothelial damage and oxidative stress (113, 114). Inhibition of Th1 differentiation in mice was shown to have atheroprotective effects by reducing IFN-γ levels in plaques (115).

Treg deficiency is associated with larger and more-advanced atherosclerotic plaques. Treg transfer into Treg-deficient models reduces inflammatory cell infiltration and decreases plaque size (116). In human carotid arteries, a higher number of Tregs inversely correlated with plaque vulnerability, suggesting their importance in maintaining plaque stability (117). This is likely related to their ability to secrete TGF-β and IL-10. In this regard, TGF-β inhibits recruitment and activation of T cells and macrophages while promoting vascular smooth muscle cell proliferation, thereby increasing plaque stability (118). IL-10 reduces IFN-γ expression by T cells, preventing T cell and macrophage recruitment and cytokine secretion (119).

The role of Th17 cells in atherosclerosis is still hotly contested (120, 121). IL-17 and IFN-γ jointly boost inflammation in atherosclerotic plaques (122). However, some studies indicated that IL-17 promotes atherosclerosis (123), while others suggest it enhances plaque stability (124, 125). Moreover, an increased Th17 cell/Treg ratio is found in patients with coronary atherosclerosis. Deletion of the leukocyte receptor CD69, which regulates Th17 cell/Treg differentiation, increases the Th17 cell/Treg ratio and exacerbates atherosclerosis in mice. Additionally, expression of CD69 mRNA in peripheral blood leukocytes from a cohort of participants with subclinical atherosclerosis correlates with a slower progression of atherosclerosis (70). This Th17 cell/Treg balance is crucial in autoimmune conditions and in mitigating adverse effects of immune checkpoint inhibitor therapy, such as myocarditis (126).

### T cell adoptive transfer.

By modulating the immune response, T cells mitigate inflammation and promote tissue repair in cardiac diseases. Therapeutic approaches that enhance Treg function or increase their numbers are being explored to reduce cardiac inflammation and fibrosis, thereby improving heart function (93, 127). Additionally, targeting specific T cell subsets — such as cytotoxic CD8^+^ T cells, which are implicated in myocarditis and autoimmune cardiac damage — offers another possible therapeutic avenue. Adoptive T cell transfer, in which patients receive their own modified T cells, and checkpoint inhibitors that regulate T cell activity are also being considered to treat arrhythmias and prevent heart tissue damage after an MI. These strategies highlight the potential of T cell–based therapies to provide targeted and effective treatments for a range of CVDs.

Cardioprotective Tregs, particularly CD4^+^ T cells reacting with α-MyHC, accumulate in the injured myocardium in humans and mice, becoming beneficial when delivered before MI in mice (78, 128). Foxp3^+^ Tregs also improve heart outcomes in mice and rats, through either autologous Treg infusion or CD28 antibody administration, which enhances Treg recruitment (129, 130). Boosting Treg accumulation in the injured heart is another promising therapeutic approach to treat human HF (131). Adoptive transfer of Tregs protected against CVB3-induced myocarditis by different mechanisms, including suppression of the immune response, fibrosis, reduction of virus titers, and improvement of cell survival via increased phosphorylation of AKT (132, 133). Adoptive transfer of Tregs inhibits the proinflammatory microenvironment of the plaque and controls the development of atherosclerosis (116). In a clinical setting, Tregs alleviate allograft rejection, but adult-derived Tregs have limitations. To overcome this, a new method uses high-quality Tregs from thymic tissue removed during pediatric cardiac surgeries (thyTregs). A phase I/II clinical trial with a 2-year follow-up of the first treated patient showed no adverse effects and conserved Treg frequency. These results support the safety and potential of autologous thyTreg therapy to restore the Treg pool in infants undergoing heart transplantation (134).

### Immune checkpoints.

A promising therapeutic avenue is targeting immune checkpoints and modulating immune responses. While immunotherapy has revolutionized oncology, its applications in CVD are underexplored (135). The CD80/86-CD28 and CD80/86–CTLA-4 immune checkpoints regulate plaque inflammation in atherosclerosis (136). CD80/86^+^ macrophages and CD28^+^ T cells are more prevalent in vulnerable plaques than in stable ones. Mice deficient in CD80 and CD86 display reduced atherosclerosis and lower IFN-γ production by effector T cells, indicating that CD28-CD80/86 interactions prime T cells in atherosclerosis (137). Mice overexpressing CTLA-4 have defective effector T cell responses and developed less atherosclerosis (138). Pharmacological inhibition of CD28-CD80/CD86 with the CTLA-4–Ig fusion protein abatacept reduced atherosclerosis in mice (139). The CD40L-CD40 interaction is a crucial immune-checkpoint target in CVD, enhancing T cell stimulation and macrophage, DC, and B cell activation. It is also essential for B cell Ig-isotype switching in germinal centers. In ApoE^–/–^ and LDLR^–/–^ mice, genetic deletion or antibody-mediated inhibition of CD40L or CD40 reduced atherosclerotic plaque burden and induced stable, collagen-rich plaques (140–142). In humans, CD40L-CD40 expression in plaques is linked to plaque vulnerability. The soluble form, sCD40L, is associated with hypercholesterolemia, stroke, diabetes, and acute coronary syndrome and can accurately predict recurrent CVD (143).

### CAR T cells.

Chimeric antigen receptors (CARs) are synthetic receptors composed of four main components: an extracellular antigen-binding domain, a hinge region, a transmembrane domain, and intracellular signaling domains. CAR T cell therapy has generated significant excitement for its ability to eradicate advanced leukemias and lymphomas, like immune checkpoint inhibitors. The use of CAR T cell therapies in CVDs is being explored, with promising preliminary results (144–146). Early studies suggest that CAR T cell–based therapies could potentially be adapted to target specific cardiovascular conditions. A therapeutic approach has been developed using modified mRNA in lipid nanoparticles (LNPs) to create transient antifibrotic CAR T cells in vivo. In a mouse model of HF, CD5-targeted LNPs efficiently delivered CAR-encoding mRNA to T cells, generating effective CAR T cells that reduced fibrosis and restored cardiac function (147). These initial findings open new avenues for treatment, offering hope for effective management and improved outcomes in patients with cardiovascular disease.

## Conclusions

**Figure 2:**
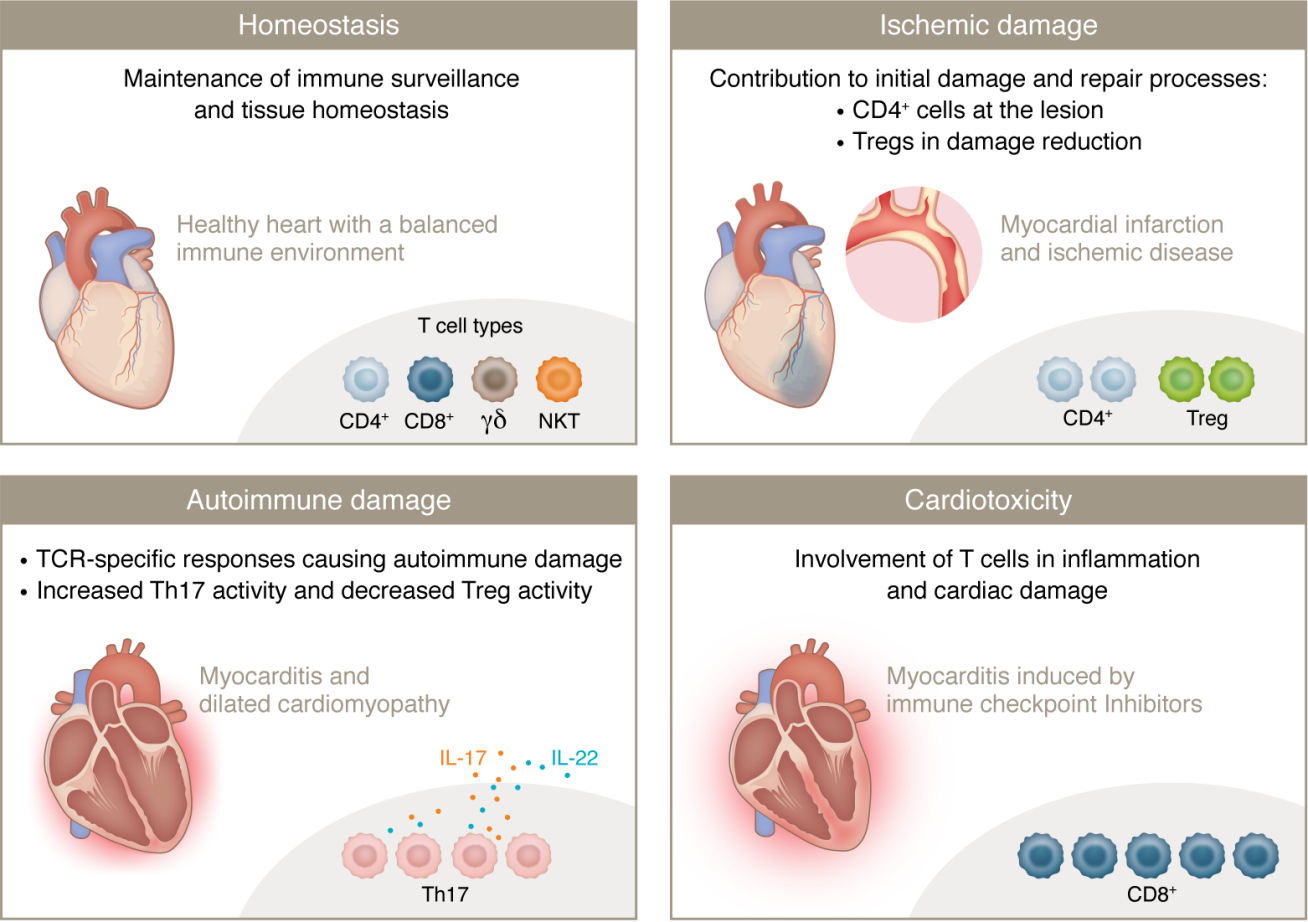
The roles of T cells in cardiac health and in different examples of disease. Homeostasis (top left): A healthy heart with a balanced immune environment. Key T cell types include CD4^+^, CD8^+^, γδ, and NKT cells. Their function is to maintain immune surveillance and tissue homeostasis. Ischemic damage (top right): A heart with myocardial infarction and ischemic heart disease. Key T cell types involved are CD4^+^ cells and Tregs. CD4^+^ T cells contribute to initial damage and repair processes, while Tregs help reduce damage. Autoimmune damage (bottom left): A heart with myocarditis and DCM. The primary T cell types are Th17 cells and Tregs. Th17 cells are involved in TCR-specific responses causing autoimmune damage, with increased Th17 activity and decreased Treg activity. Cardiotoxicity (bottom right): A heart affected by myocarditis induced by immune checkpoint inhibitors. The main T cell type involved is CD8^+^ T cells, which play an important role in inflammation and cardiac damage.

The pivotal role of T cells in CVD underscores their dual function in both maintaining cardiac health and contributing to pathology. T cells are integral to immune system–mediated regulation of cardiac inflammation, repair, and remodeling. Their diverse subsets, including Th1 cells, Th17 cells, Tregs, and cytotoxic T cells, play specific roles in myocardial inflammation, ischemic damage, and autoimmune responses (Figure 2).

Emerging therapies targeting T cells — such as immune checkpoint inhibitors, Treg adoptive transfer, and CAR T cell therapies — show promise in modulating immune responses and improving clinical outcomes in CVD patients: (a) Immune checkpoint inhibitors, initially developed for oncology, are being explored for their capacity to reduce atherosclerotic plaque burden and enhance plaque stability; (b) Treg therapy aims to increase the population of these regulatory cells to suppress harmful immune responses and promote healing in the heart; and (c) CAR T cell therapy, although primarily used in cancer treatment, is being investigated for its potential to target specific antigens involved in cardiac fibrosis and inflammation.

These innovative approaches are not only enabling us to understand the mechanisms of T cell interactions within the cardiac microenvironment but serve as early examples of targeted and effective immunotherapeutic strategies. Ultimately, this will enhance our ability to prevent, manage, and treat various CVDs, leading to improved patient outcomes and quality of life. The crossroad of immunology and cardiology opens a promising frontier for novel therapeutic interventions, offering hope for more precise and effective treatments for cardiovascular conditions.

## Version 1 01/16/2025

Electronic publication
